# Centrality in Complex Networks with Overlapping Community Structure

**DOI:** 10.1038/s41598-019-46507-y

**Published:** 2019-07-12

**Authors:** Zakariya Ghalmane, Chantal Cherifi, Hocine Cherifi, Mohammed El Hassouni

**Affiliations:** 1 LRIT, URAC No 29, Rabat IT Center, University Mohammed V, Rabat, Morocco; 2 DISP Lab, University of Lyon 2, Lyon, France; 3 LIB EA 7534, University of Burgundy, Dijon, France

**Keywords:** Computer science, Applied mathematics

## Abstract

Identifying influential spreaders in networks is an essential issue in order to prevent epidemic spreading, or to accelerate information diffusion. Several centrality measures take advantage of various network topological properties to quantify the notion of influence. However, the vast majority of works ignore its community structure while it is one of the main features of many real-world networks. In a recent study, we show that the centrality of a node in a network with non-overlapping communities depends on two features: Its local influence on the nodes belonging to its community, and its global influence on the nodes belonging to the other communities. Using global and local connectivity of the nodes, we introduced a framework allowing to redefine all the classical centrality measures (designed for networks without community structure) to non-overlapping modular networks. In this paper, we extend the so-called “Modular Centrality” to networks with overlapping communities. Indeed, it is a frequent scenario in real-world networks, especially for social networks where nodes usually belong to several communities. The “Overlapping Modular Centrality” is a two-dimensional measure that quantifies the local and global influence of overlapping and non-overlapping nodes. Extensive experiments have been performed on synthetic and real-world data using the Susceptible-Infected-Recovered (SIR) epidemic model. Results show that the Overlapping Modular Centrality outperforms its alternatives designed for non-modular networks. These investigations provide better knowledge on the influence of the various parameters governing the overlapping community structure on the nodes’ centrality. Additionally, two combinations of the components of the Overlapping Modular Centrality are evaluated. Comparative analysis with competing methods shows that they produce more efficient centrality scores.

## Introduction

Finding influential nodes in a complex network is a core question for researchers^1–6^. It has a plethora of applications in different domains, such as the promotion of commercial products, the spread of news and ideas and the outbreak of a disease. Up to now, different centrality measures tied to the network topology have been introduced to solve the issue of finding these key nodes^7^. Among the various topological properties, many real-world networks^8–11^ exhibit a modular organization. Indeed, these networks are made of firmly connected groups of nodes, where connections across modules are relatively sparse. The modules can share some nodes or not. Indeed, in networks with overlapping communities, some nodes belong to several communities, while in networks with non-overlapping communities all the nodes belong to a single community. Despite the importance of the community structure, most of the proposed centrality measures ignore this property. Few works devoted to networks with non-overlapping community structure^1–6,12–23^ and overlapping community structure^24–28^ have shown that it can be efficiently exploited in order to produce efficient centrality scores. Indeed, all these works highlight the influence of the modular organization on the diffusion process.

In a recent work^29^, we introduced a framework to adapt centrality measures proposed for networks with no community structure to non-overlapping modular networks. The “Modular Centrality” is a vector where each dimension accounts for a different type of influence that the nodes can exert in the network. The first dimension quantifies their local influence in their own community, while the second dimension measures their global influence on the other nodes to which they are linked outside of their community. Extensive analysis of Modular Centrality using synthetic and real-world data have demonstrated its superiority over both classical measures and alternative methods designed for modular networks.

In this study, we extend this framework to networks with overlapping modules. Indeed, overlapping nodes introduce a new challenge. As they can belong to multiple communities, we need to redefine the local and global notions of influence, especially for the overlapping nodes. In networks with overlapping community structure, one needs to consider also the global and local influence of the nodes. However, the local influence depends on the type of nodes(overlapping, non-overlapping). Through the intra-community links, the non-overlapping nodes exert a local influence on the nodes belonging to their communities, while the overlapping nodes exert a local influence on nodes of all the communities to which they belong. In addition, both types of nodes have a global influence on nodes that belong to different neighboring communities through the inter-community links. Inspired by this idea, we propose the so-called “Overlapping Modular centrality”, which is a new representation of the standard centrality measures dedicated to networks with overlapping community structure. It is a two-dimensional vector, where each component stands for the different types of influence. Based on the most influential centrality measures (Degree centrality, Betweenness centrality, Closeness centrality, Eigenvector centrality), we perform a comparative analysis of the local and global component with their classical counterpart designed for non-modular networks. Furthermore, we propose and investigate two ranking measures based on combining the local and global components (the modulus of the Overlapping Modular Centrality vector and a weighted linear combination of both components). As there are multiple ways to combine both components, rather than concentrating on the most efficient ranking method, we investigate the gains in performances that can be obtained using additional knowledge about the topology of the communities.

Given a classical centrality measure designed for non-modular networks, to compute its overlapping modular version one has to proceed as follows:

- Extract the local network from the original modular network.
- Extract the global network from the original modular network.
- Compute the global component of the Overlapping Modular Centrality on the global network.
- Compute the local component of the Overlapping Modular Centrality on the local networks.

To evaluate the effectiveness of the various centrality measures, we consider an epidemic process setting. The classical SIR model is used in order to simulate and investigate the spread of diseases in the network. Tests are performed on synthetic networks generated by the LFR algorithm^30^ and real-world networks. Experimental results point out that the Overlapping Modular Centrality always outperforms their standard counterparts defined for networks without community structure. Additionally, performances increase when the number of overlapping nodes or the number of communities to which they belong (their membership degree) increase.

Defining the set of communities may give a clear idea of how the network is organized. We can then distinguish between nodes acting as hubs inside the communities and nodes located at the boundaries making the epidemics spread to other modules. Community detection is one of the most challenging issues in complex network. The cohesion and separation of communities are considered differently by the formal definitions. Thus, there is no universal definition on the modules that one need to look for. This ambiguity leads to the proposal of different community detection algorithms using the notion of the community structure differently. Indeed, some algorithms consider that nodes belong to only one single community while others consider that nodes may belong to several communities which form the overlaps between the modules. In this paper, we use two overlapping community detection algorithms to unveil the set of communities of the network. The Speaker-Listener Label Propagation Algorithm (SLPA) as well as Link Communities (LINKC) algorithms are employed to compute the Overlapping Modular centrality. The results show that the Overlapping Modular Centrality is at its best when SLPA is used. These experiments show that the choice of the community detection algorithm can impact the performance of the Overlapping Modular Centrality. Moreover, the Louvain algorithm is also used to compare the Overlapping Modular centrality with the Modular centrality since Louvain algorithm is designed for non-overlapping communities. Results show that the SLPA and LINKC detection algorithms present a better performance as compared to Louvain algorithm whatever the community structure strength. Therefore, the overlapping nodes have a crucial impact on the epidemic spreading process.

## Overlapping Modular Centrality Model

In this section, we present the different elements that make up the basis of the proposed approach. The Overlapping Modular Centrality definition and the algorithm to compute its components is given.

In networks with an overlapping community structure, the influence of nodes can be decomposed in two parts: A local influence associated to the interactions with the members of their communities, and a global influence related to their interactions with nodes from neighboring communities. Based on this assumption, we propose the Overlapping Modular Centrality.

In order to compute these two components, the original network is decomposed into a local network that captures the interactions inside the communities and a global network that accounts for the interactions between the communities. The local network definition depends on the type of node that is considered. For a non-overlapping node, it reduces to its unique community, while for an overlapping node, it includes all the communities to which it belongs.

Let’s $G(V,E)$ be a simple undirected network. $V={V}_{o}\cup {V}_{no}$ is the set of nodes, where *V*_*o*_ and *V*_*no*_ represent the set of overlapping and non-overlapping nodes respectively, and *E* is the set of edges. ${\mathscr{C}}=\{{C}_{1},\mathrm{..}\,{C}_{k}.,{C}_{m}\}$ is the set of communities and *m* is the number of communities.

### Definitions

#### Local component of the overlapping modular centrality

The local neighborhood of an overlapping node *M*_*y*_ is defined as the union of the communities to which it belongs ${M}_{y}={\cup }_{r=1}^{s}\,{C}_{r}$. The local network *G*_*l*_ is the set formed by the union of the local neighborhoods ${G}_{l}={\cup }_{y=1}^{z}\,{M}_{y}$, where ${\mathcal M} =\{{M}_{1},\mathrm{..}{M}_{y}\mathrm{..},{M}_{z}\}$ is the set of the obtained connected components and $z=| {\mathcal M} |$ is the size of the set ${\mathcal M}$. Each component *M*_*y*_ is denoted as ${M}_{y}({V}_{y},{E}_{y})$. Where ${V}_{y}=\{{v}_{i}^{y}\backslash {v}_{i}\in V\}$ and ${E}_{y}=\{({v}_{i}^{{y}_{1}},{v}_{j}^{{y}_{2}})\backslash {v}_{i},{v}_{j}\in V$ and ${y}_{1}={y}_{2}\}$, while ${v}_{i}^{y}$ refers to any node *v*_*i*_ belonging to the component *M*_*y*_. For a given centrality measure *β*, we define ${\beta }_{L}({v}_{i}^{y})$ as the local component of the Modular Centrality of the overlapping node ${v}_{i}\in {M}_{y}$. It is computed over all the connected components of the local network *G*_*l*_.

A non-overlapping node is a special case of an overlapping one. This type of nodes belongs to only one community *C*_*k*_. In order to compute the local component of a non-overlapping node, we define the local network denoted as *G*_*l*_ as the union of all the modules of the network ${G}_{l}={\cup }_{k=1}^{m}\,{C}_{k}$. These isolated communities are obtained by removing all the inter-community links from the original network *G*, and by replicating all the overlapping nodes with their intra-community links in each of their shared communities. Each module represents a community *C*_*k*_ denoted as ${C}_{k}({V}_{k},{E}_{k})$, where ${V}_{k}=\{{v}_{i}^{k}\backslash {v}_{i}\in V\}$ and ${E}_{k}=\{({v}_{i}^{{k}_{1}},{v}_{j}^{{k}_{2}})\backslash {v}_{i},{v}_{j}\in V$ and ${k}_{1}={k}_{2}=k\}$, while ${v}_{i}^{k}$ refers to any node *v*_*i*_ belonging to the community *C*_*k*_. Let’s consider a non-overlapping node ${v}_{i}^{k}\in {C}_{k}$ and a given centrality measure *β*. Its local centrality component ${\beta }_{L}({v}_{i}^{k})$ is computed in its local neighborhood (its isolated community) *C*_*k*_ using the usual definition of *β*.

#### Global component of the overlapping modular centrality

The global network *G*_*g*_ is defined as the union of all the connected components of the graph remaining after deleting all the intra-community links in the original network and all the isolated nodes. ${G}_{g}={\cup }_{q=1}^{p}\,{S}_{q}$, where ${\mathscr{S}}=\{{S}_{1},\mathrm{..}{S}_{q}\mathrm{..},{S}_{p}\}$ is the set of connected components and $p=|{\mathscr{S}}|$ is the size of the set ${\mathscr{S}}$. Each component ${S}_{q}$ is denoted as ${S}_{q}({V}_{q},{E}_{q})$, where ${V}_{q}=\{{v}_{i}^{q}\backslash {v}_{i}\in V\}$ and ${E}_{q}=\{({v}_{i}^{{q}_{1}},{v}_{j}^{{q}_{2}})\backslash {v}_{i},{v}_{j}\in V$ and ${q}_{1}={q}_{2}\}$, while ${v}_{i}^{q}$ refers to any node *v*_*i*_ belonging to the component *S*_*q*_.

For a given centrality measure *β*, we define ${\beta }_{G}({v}_{i}^{q})$ as the global component of the overlapping modular centrality of the node ${v}_{i}\in {S}_{q}$. It is computed over all the components *S*_*q*_ of the global graph *G*_*g*_. The global component of the overlapping modular centrality of isolated nodes is set to zero.

#### Overlapping modular centrality

Given a centrality measure *β*, designed for non modular networks (Betweeness, Degree, etc.), its Overlapping Modular extension for a node ${v}_{i}\in V$ is expressed as follows:

1 $${B}_{OM}({v}_{i})=({\beta }_{L}({v}_{i}^{y}),{\beta }_{G}({v}_{i}^{q}))\,y\in \{1,\ldots ,z\}\,{\rm{and}}\,q\in \{1,\ldots ,p\}$$

where *β*_*L*_ and *β*_*G*_ stand respectively for the Local and the Global components of the Overlapping Modular centrality of a node *v*_*i*_. Note that the global and local network extracted are not subnetworks of the original modular network. They are sets of independent connected components build with replication of some of the nodes and links of the original networks.

## Algorithm

The overlapping modular centrality calculation procedure may be specified as follows:

**Step 1**. Choose a centrality measure defined for non modular networks *β*.

**Figure 1:**
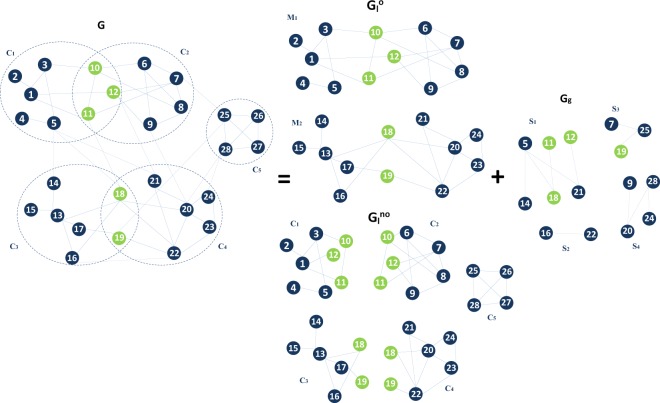
Toy example to illustrate how to form the local network for the non-overlapping nodes $({G}_{l}^{no})$, the local network for the overlapping nodes $({G}_{l}^{o})$ and the Global network $({G}_{g})$ from a network $(G)$ with an overlapping community structure.

**Step 2**. Build the local network of the overlapping nodes *G*_*l*_ defined as the set obtained by the union of the local neighborhoods as it is illustrated in Fig. 1.

**Step 3**. For each module ${M}_{y}\in  {\mathcal M}$, compute the local component *β*_*L*_ for all the overlapping nodes ${v}_{i}^{y}\in {V}_{y}\backslash {V}_{no}$.

**Step 4**. Delete the inter-community links from *G*, and replicate all the overlapping nodes with their intra-community links in each of their shared communities to build the local network for non-overlapping nodes as it is illustrated in Fig. 1.

**Step 5**. For each community ${C}_{k}\in {\mathscr{C}}$, compute the local component *β*_*L*_ of all the non-overlapping nodes ${v}_{i}^{k}\in {V}_{k}\backslash {V}_{o}$.

**Step 6**. Delete the intra-community links and the isolated nodes in order to build the global network *G*_*g*_ as it is shown in Fig. 1.

**Step 7**. For each module ${S}_{q}\in {\mathscr{S}}$, compute the global component of the Overlapping Modular Centrality *β*_*G*_ of all the nodes ${v}_{i}^{q}\in {V}_{q}$. The global centrality of isolated nodes is set to 0.

**Step 8**. Add *β*_*L*_ and *β*_*G*_ to the Overlapping Modular centrality vector *B*_*OM*_.

### General scheme

**Figure 2:**
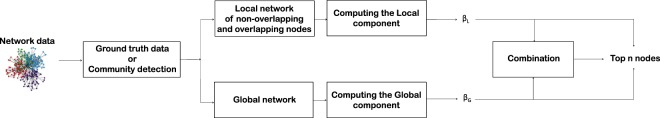
The main steps of the proposed ranking framework.

Figure 2 illustrates the nodes ranking methodology in networks with overlapping community structure. First of all, one needs to know the community structure. If there is no ground-truth data available, a community detection algorithm^31,32^ can be used to uncover the community structure. Given the community structure, the local and global networks are extracted. Remember that the local network of the overlapping nodes is formed by the union of the local neighborhoods. The local network of the non-overlapping nodes is built by deleting all the inter-community links from the original network and by replicating all the overlapping nodes with their intra-community links in each of their shared communities. In addition, the global network is the result of deleting all the intra-community links together with all the remaining isolated nodes. Consider a centrality measure such as the Degree centrality, for example, its local component of the Overlapping Modular Centrality is computed on the local network, while its global component is computed on the global network. Nodes that are not in the global network are assigned a null global centrality value. Finally, the top influential nodes are selected according to their rank. They can be ranked according to the local or global component of the Overlapping Modular centrality or to a measure based on their combination (refer to the Materials and Methods section for the definitions of the ranking measures derived from a combination of both components).

## Results on Synthetic Networks

The aim of this set of experiments is to evaluate the influence of the various parameters of the communities (the community structure strength, the proportion of the overlapping nodes in the network and their community membership degree). They are performed in an epidemic spreading setting based on the SIR Model on LFR generated synthetic networks. LFR allows generating networks with overlapping community structure. The community structure strength can be adjusted via the mixing parameter *μ*. It represents the fraction of the inter-community links in networks and ranges from 0 to 1. It is set to a small value to generate networks with a well-defined community structure (few edges between communities). The mixing parameter is set to a large value to generate networks with a loose community structure. In these networks, nodes have more connections with nodes in other communities than with the ones in their communities. Moreover, networks with medium community structure strength can be generated with *μ* values ranging from 0.2 to 0.35. The degree and community size distributions of the networks follow a Power law with a tunable exponent value. Other parameters such as *on* and *om* can control the proportion of the overlapping nodes in the network and their membership respectively. We compare the ranking effectiveness associated with the Overlapping Modular Centrality extensions of Degree, Betweenness, Closeness and Eigenvector centrality with those obtained using their traditional counterpart.

### Influence of the community structure strength

**Figure 3:**
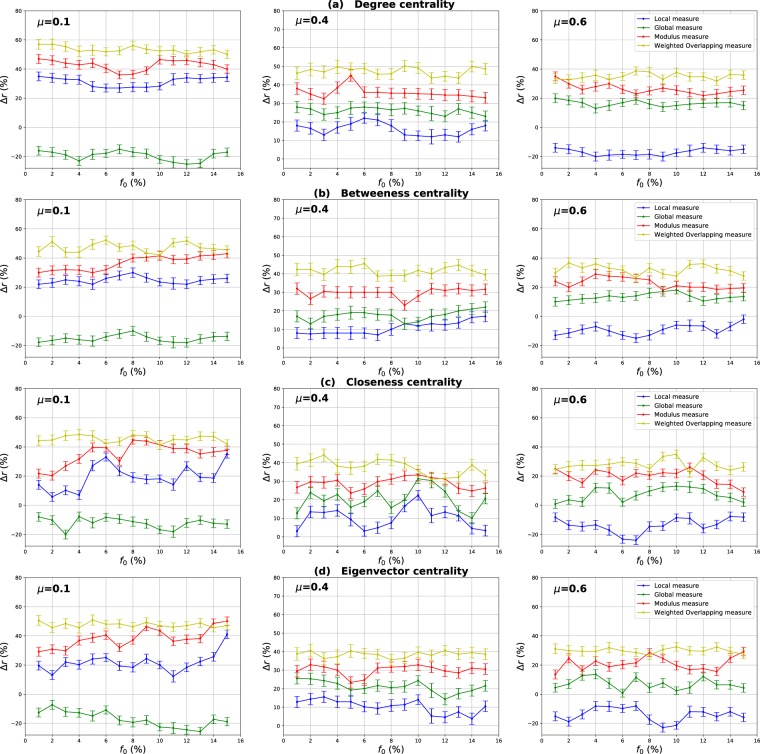
Relative difference of the outbreak size Δ*r* versus the percentage of initial spreaders *f*_0_, where ${\rm{\Delta }}r=({R}_{c}-{R}_{s}/{R}_{s})$, *R*_*c*_ and *R*_*s*_ are the final number of recovered nodes for the centrality measure under test and the standard centrality, respectively. The Degree (**a**), Betweenness (**b**), Closeness (**c**) and Eigenvector (**d**) centrality measures are compared to their extensions derived from the Overlapping Modular Centrality. Synthetic networks generated by the LFR algorithm with different community structure strength are used (the value of their mixing coefficient is equal to 0.1, 0.4 and 0.6). We set the proportion of overlapping nodes *on* to 10% of the size of the network and the community membership *om* to 10% of the total number of communities. Each value on the curves is obtained by averaging the results of 200 SIR simulations per method and fraction of initially infected nodes. Δ*r* is positive if the centrality under test is more effective than the standard centrality.

The mixing parameter value *μ* controls the percentage of inter-community links and therefore the community structure strength ($0 < \mu  < 1$). For low values of *μ*, networks exhibit a well-defined community structure (few inter-community links). Increasing the proportion of inter-community links blurs the community structure. In order to study the influence of the community structure strength on the performance of the various centrality measures, networks with three mixing parameter values ($\mu =0.1,0.4,0.6$) have been generated and SIR simulations have been performed. The curves in Fig. 3 report the relative difference of the outbreak size Δ*r* versus the fraction of initial spreaders obtained with the standard measure ((a) Degree centrality (b) Betweenness centrality (c) Closeness centrality (d) Eigenvector centrality) used as a reference. Positive values indicate that the standard measure is less effective, while it is the contrary for negative values.

#### Evaluation of the local and the global component of the overlapping modular centrality

Results for networks with well-defined community structure ($\mu =0.1$) are reported on the left panel of Fig. 3. In this case, the local component of the Overlapping Modular Centrality always outperforms the standard measure. The gain on the standard measure is around 25% for Degree centrality, and it is around 22% for Betweenness centrality. The smallest gain is for Closeness and Eigenvector centrality measures with an average value of 20%. However, the classical measures are more effective than the global component of the Overlapping Modular centrality. In this situation, there are few connections between the communities, and it is more appropriate to immunize nodes with high local influence inside their communities. Indeed, since there are few inter-community edges, if an epidemic initiates in the core of a community, it may die before propagating to the periphery of the community and the other parts of the network. Therefore, in networks with well-defined community structure, it is a better strategy to target nodes with high local influence than isolating the communities.

The middle panel of f1 presents the results for networks with medium community structure strength ($\mu =0.4$). In these figures, Δ*r* is always positive for both components of the Overlapping Modular Centrality. The local component of the Overlapping Modular Centrality performs better than the standard measure with a gain of an average value of 10% for Betweenness, Closeness and Eigenvector centrality. The largest gain is for the Degree centrality with an average value of 15%. The global measure outperforms the standard measure with a gain of around 18% for Betweenness centrality, 20% for Closeness and Eigenvector centrality. The largest gain is also for Degree centrality with an average value of 28%. Therefore, the global measure overall is more accurate than the local measure. In this situation, there are almost as many intra-community links as there are inter-community links. Thus, if an epidemic initiates in a community, it may easily propagate to the various communities through the numerous inter-community links. This is why the global influence is more relevant than the local influence in networks with community structure of average strength.

Results of the experiments using networks with a weak-community structure ($\mu =0.6$) are reported in the right panel of Fig. 3. These curves show that the relative difference of the outbreak size Δ*r* is positive or the global component, while it is always negative for the local component of the Overlapping Modular Centrality. The average gain on the traditional Betweenness, Closeness and Eigenvector centrality measures is around 10% for the global component of the Overlapping Modular Centrality. The largest gain is still reached for Degree centrality with an average value of 15%. The local component of the Overlapping Modular Centrality performs worse than the standard measure with an average value of 15% for Degree, Betweenness, Closeness and Eigenvector centrality measures. The inter-community links predominate in this type of networks. Therefore, these edges can disseminate epidemics globally all over the network. The best strategy in this situation is to isolate the communities.

#### Evaluation of the ranking methods based on combining the components of the overlapping modular centrality

In order to evaluate the impact of combining local and global information, we consider two ranking measures. The first one is the modulus of both components of the Overlapping Modular centrality. The second one is a weighted linear combination of the two components. The weights are computed using more knowledge about the communities (community size, number of neighboring communities). We expect that using additional community structure parameters, the effectiveness of the combination increases. In Fig. 3, we report the experimental results of the ranking measures based on the modulus and the weighted linear combination of the local and the global component of the Overlapping Modular Centrality. It appears clearly that they outperform the classical measure and also each component of the Overlapping Modular Centrality. This result is valid for all the centrality measures under test. Indeed, the modulus performs better than the standard measure with an average gain of 37% in networks with strong community structure, 30% in networks with community structure of medium strength, and 20% in networks with weak community structure for Betweenness, Closeness, and Eigenvector centrality measures. The largest gain is for Degree centrality with an average value of 42% in networks with strong community structure, 35% in networks with community structure of medium strength, and 25% in networks with weak community structure. Therefore, combining the components of the Overlapping Modular Centrality is much more efficient than using only one of its components or the classical centrality measure.

Furthermore, one can notice on Fig. 3 that the curves of the Weighted linear combination of the Overlapping Modular centrality components are located in the top in all the figures. It is, therefore, the most effective measure whatever the community structure strength. Actually, the gain is around 47% in networks with well-defined community structure, 40% in networks with community structure of medium strength and 30% in networks with loose community structure for Betweenness, Closeness and Eigenvector centrality. Degree centrality presents the largest gain with an average value of 52% in networks with well-defined community structure, 45% in networks with community structure of medium strength and 35% in networks with loose community structure. This confirms our intuition that further gain can be obtained by using additional knowledge about the topological properties of the community structure. Indeed, in this measure, we introduce the fraction of inter-community links of each community in the case of non-overlapping nodes, and of each local neighborhood module in the case of overlapping nodes. The idea is to target the most influential nodes in networks taking care of each community topological properties. We introduce also the size of the communities in order to target hubs in large communities for their high local influence. In addition, the number of neighboring communities is also used to select community bridge nodes connected to multiple communities. Indeed, their global influence is high. Adjusting the weights allow adapting to the community structure by favoring either the local component or the global component. As this measure incorporates additional information about the community structure, it is more efficient than the modulus, the local and the global components of the Overlapping Modular Centrality. To summarize, using a combination strategy is more accurate as compared to the local, global or traditional measures. These results are obtained for different centrality measures and for networks with various community structure strength. The best gains are obtained in networks with a well-defined community structure. Indeed, increasing the mixing parameter value blurs the community structure. The network tends to act more and more like a single big community. Consequently, the modular-based measures performance decrease. As expected, better results are obtained by using more relevant information about the topological properties of the community structure. These results suggest that further improvements can be reached using even more efficient combination strategies.

### Influence of the number of overlapping nodes

**Figure 4:**
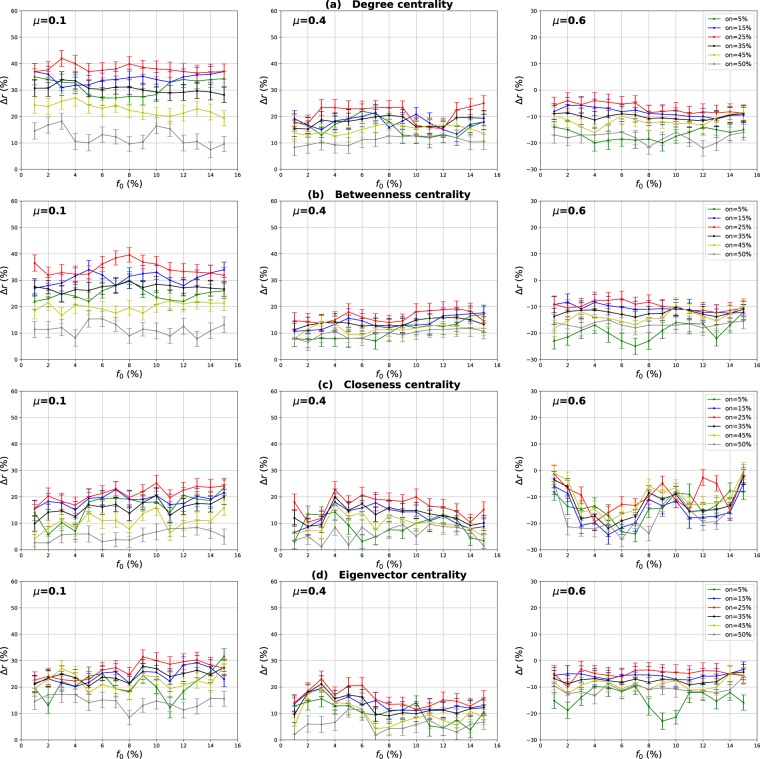
Influence of the number of overlapping nodes. The relative difference of the outbreak size Δ*r* between the standard measure and the Local measure versus the percentage of initial spreaders *f*_0_. The community membership value *om* is equal to 10% of the total number of communities.

**Figure 5:**
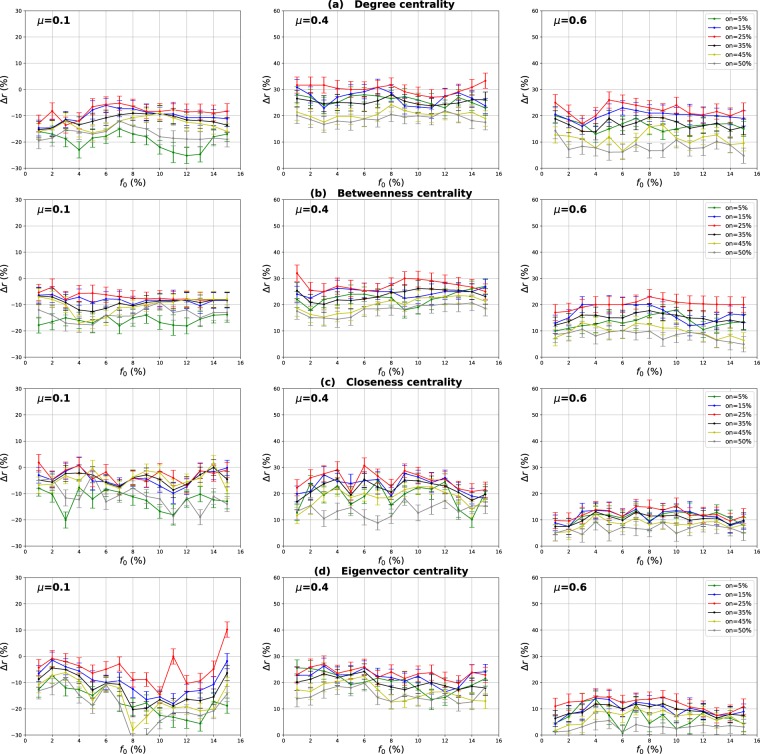
Influence of the number of overlapping nodes. The relative difference of the outbreak size Δ*r* between the standard measure and the Global measure versus the percentage of initial spreaders *f*_0_. The community membership value *om* is equal to 10% of the total number of communities.

**Figure 6:**
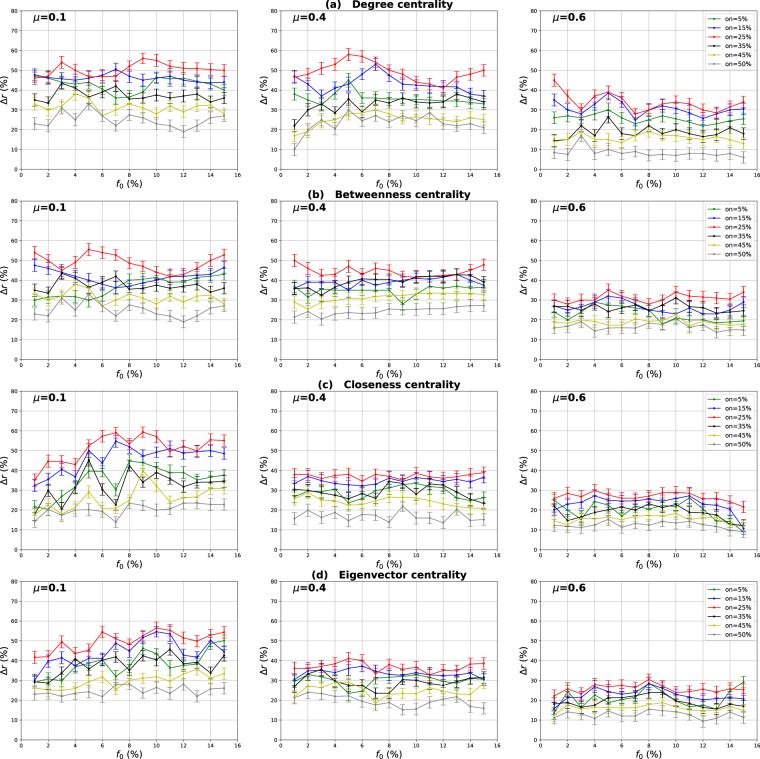
Influence of the number of overlapping nodes. The relative difference of the outbreak size Δ*r* between the standard measure and the modulus measure versus the percentage of initial spreaders *f*_0_. The community membership value *om* is equal to 10% of the total number of communities.

**Figure 7:**
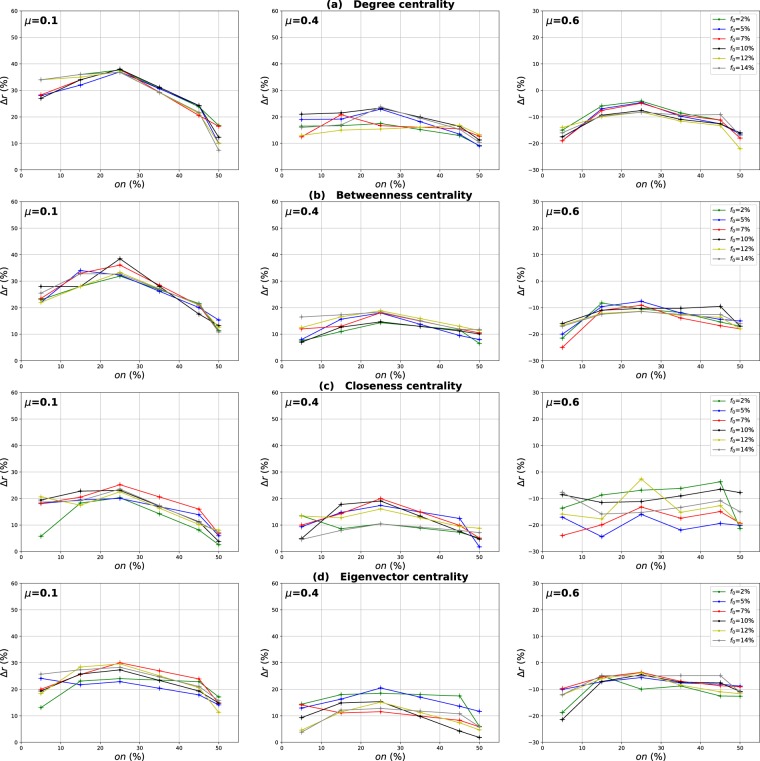
Influence of the number of overlapping nodes. The relative difference of the outbreak size Δ*r* between the standard measure and the Local measure versus the percentage of the overlapping nodes *on*. The community membership value *om* is equal to 10% of the total number of communities.

**Figure 8:**
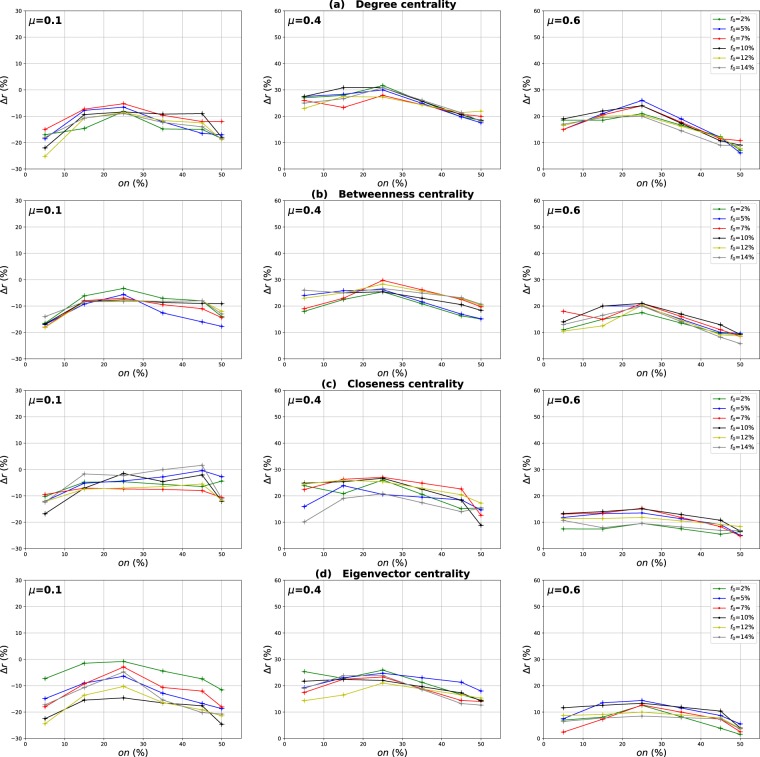
Influence of the number of overlapping nodes. The relative difference of the outbreak size Δ*r* between the standard measure and the Global measure versus the fraction of the overlapping nodes *on*. The community membership value *om* is equal to 10% of the total number of communities.

**Figure 9:**
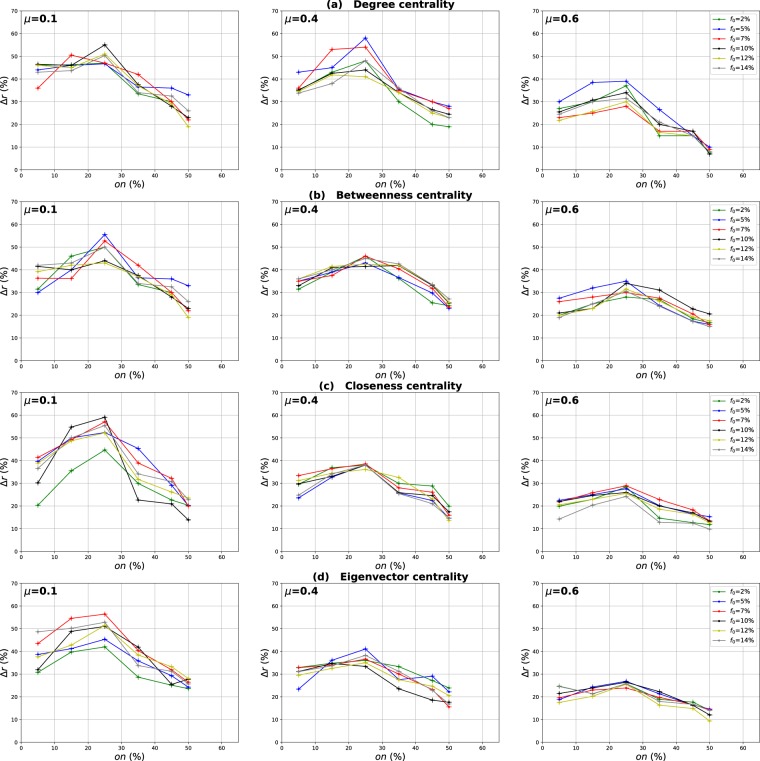
Influence of the number of overlapping nodes. The relative difference of the outbreak size Δ*r* between the standard measure and the modulus measure versus the percentageof the overlapping nodes *on*. The community membership value *om* is equal to 10% of the total number of communities.

The aim of this investigation is to show the influence of the proportion of the overlapping nodes on the performance of the Overlapping Modular Centrality. Networks with different community structure strength are used in this experiment (the mixing parameter is ranging from $\mu =0.1$ to $\mu =0.6$). In addition, the value of the community membership degree is set to 10% of the total number of the communities found in each network. The relative difference of the outbreak size Δ*r* of the local, global and the modulus measures versus the fraction of initial spreaders *f*_0_ is represented in Figs 4–6. It is represented as a function of the proportion of the overlapping nodes *on* in Figs 7–9.

One can see in Figs 4 and 5 that the local and the global measures exhibit the same behavior if the proportion of overlapping nodes changes. Actually, their performance increase as the proportion of the overlapping nodes increases. These results are valid for all the centrality measures as it is illustrated in Figs 7 and 8. However, their performance decreases when the proportion of the overlapping nodes exceeds an average value of 27% as it is shown in Figs 7 and 8. The performance of the local measure for instance in networks with strong community structure raises with an average gain of 11% for Degree and Betweenness centrality, and 7% for Closeness and Eigenvector centrality measures. The average gain is around 6% in networks with medium community structure strength for all the centrality measures, as it is reported in Fig. 7. The performance of the global measure increases with an average gain of 5% in networks with strong community structure, 4% in networks with community structure of medium strength. The biggest gain is obtained with Degree and Betweenness centrality in networks with non-cohesive community structure. Indeed, in this case, we observe an average gain of 7%, while it is 3% with Closeness and Eigenvector centrality as illustrated in Fig. 8.

Increasing the proportion of the overlapping nodes, the size of their local network grows. Consequently, their local influence increase, since these nodes can belong to several neighboring communities. This is why the ranking based on the local component of the Overlapping Modular Centrality becomes more efficient. Moreover, when the proportion of overlapping nodes becomes larger, there is a great chance that the proportion of the overlapping nodes inside the global network gets also bigger. Thus, these nodes can disseminate the epidemic globally in many foreign communities. This, therefore, increases the global influence of these nodes in the network. Hence, the performance of the global measure increases while increasing the proportion of the overlapping nodes. Furthermore, when the proportion of the overlapping nodes becomes very important, the community structure becomes loose since the majority of nodes can belong to several communities. Consequently, the performance of the local and the global components decrease when the proportion of the overlapping nodes exceeds the average value of 27%.

The same kind of results are shown in Figs 6 and 9 for the modulus based measure. In Fig. 6, one can notice that the modulus measure exhibits the same behavior in networks with different community structures. Its performance increases as the proportion of overlapping nodes increases. Yet, its performance stops increasing when the proportion of the overlapping nodes exceeds an average value of 27%. As it is shown in Fig. 9, this is true for networks with various community structure strength and for all the centrality measures. The modulus based measure performance increases by an average gain of 13% in networks with clear community structure, 10% in networks with medium community structure strength, and 8% in networks with unclear community structure. this behavior is quite independent of the centrality measures as it is reported in the left, middle and right panel of Fig. 9. As expected, the increase of the local and the global influence of nodes leads also to an increase in the performance of the modulus measure. This is due to the fact that this ranking measure is based on a combination of local and global components of the Overlapping Modular Centrality.

### Influence of the overlapping community membership degree

**Figure 10:**
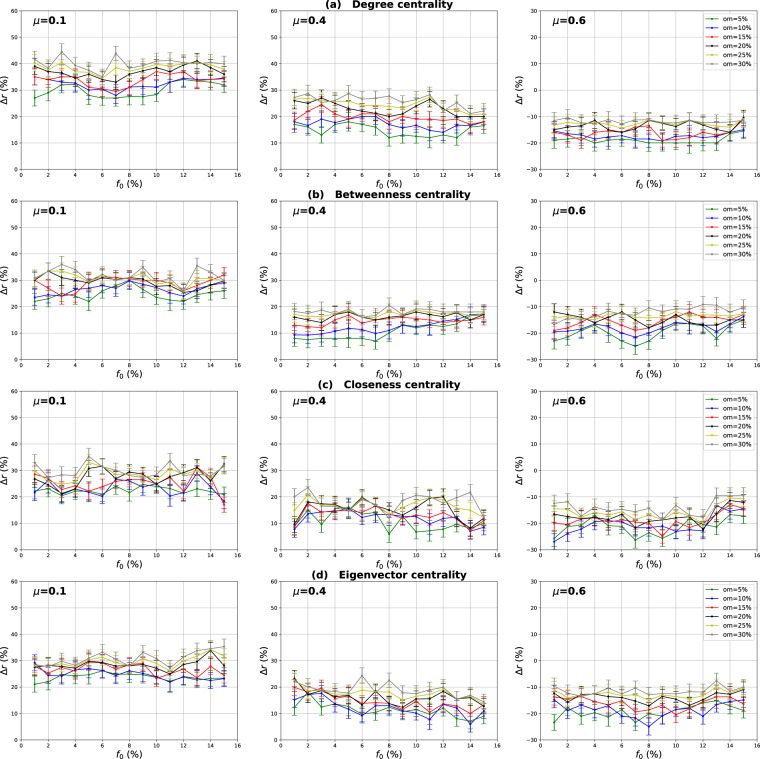
Influence of the community membership degree. The relative difference of the outbreak size Δ*r* between the standard measure and the Local measure versus the percentage of initial spreaders *f*_0_. The number of overlapping nodes value *on* is equal to 10% of the total number of the nodes.

**Figure 11:**
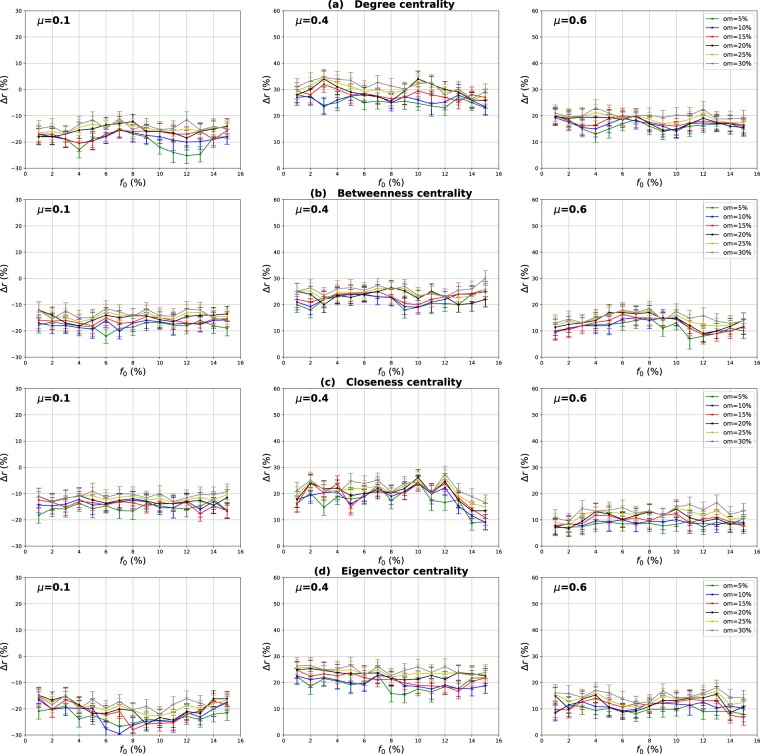
Influence of the community membership degree. The relative difference of the outbreak size Δ*r* between the standard measure and the Global measure versus the percentage of initial spreaders *f*_0_. The number of overlapping nodes value *on* is equal to 10% of the total number of the nodes.

**Figure 12:**
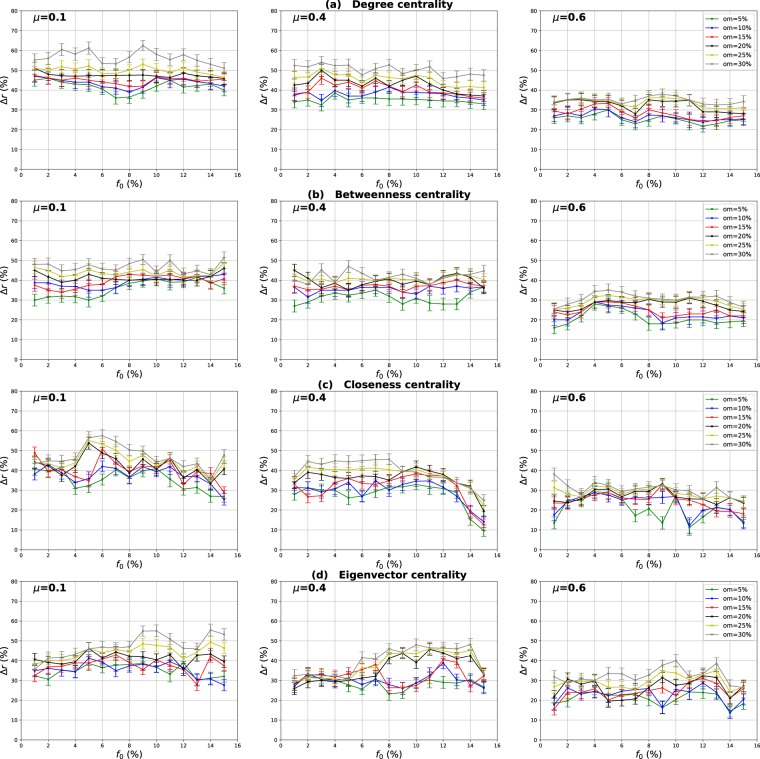
Influence of the community membership degree. The relative difference of the outbreak size Δ*r* between the standard measure and the Modulus measure versus the percentage of initial spreaders *f*_0_. The number of overlapping nodes *on* is equal to 10% of the total number of the nodes.

**Figure 13:**
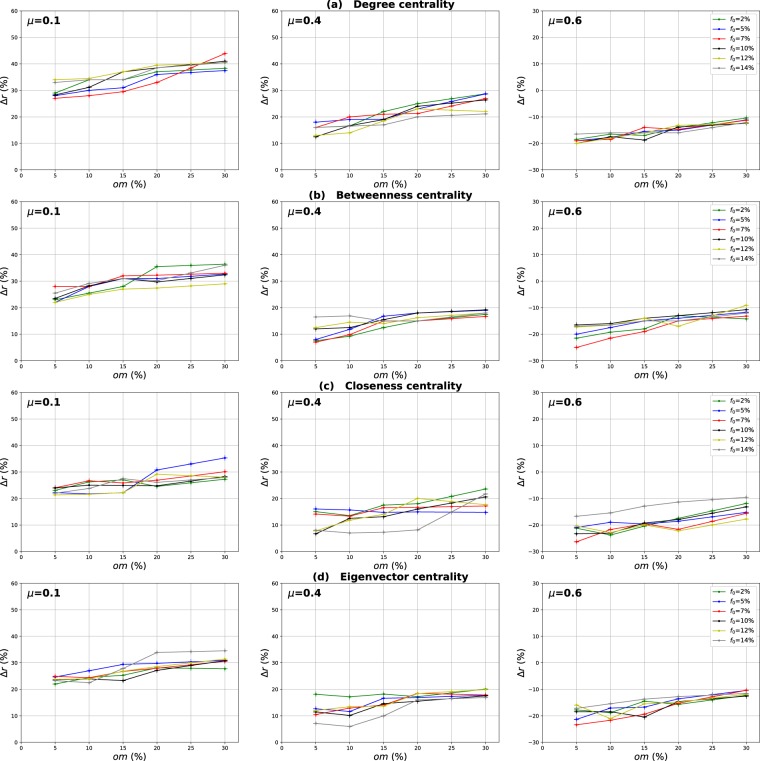
Influence of the community membership degree. The relative difference of the outbreak size Δ*r* between the standard measure and the Local measure versus the membership degree *om*. The number of overlapping nodes value *on* is equal to 10% of the total number of communities.

**Figure 14:**
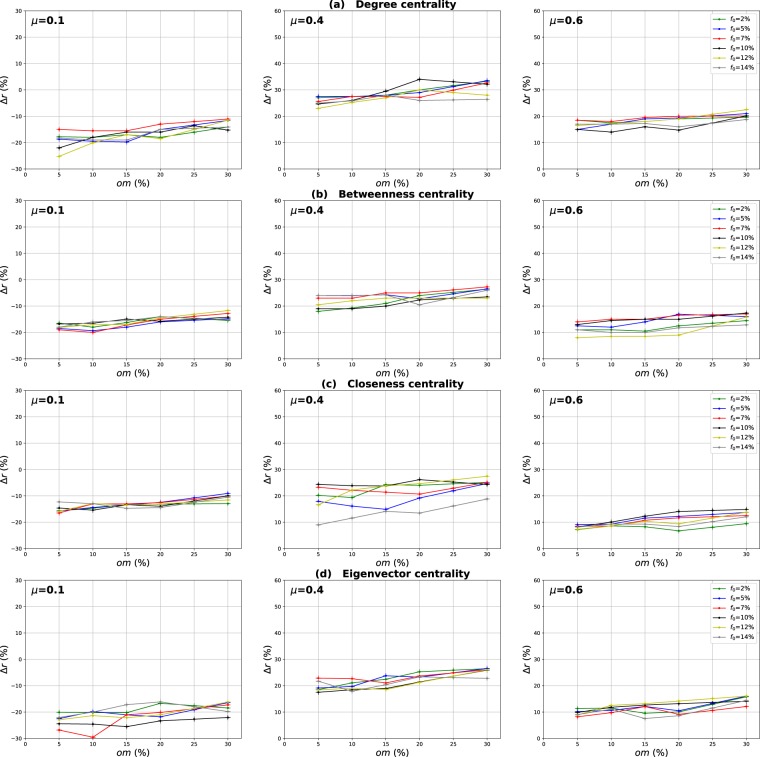
Influence of the community membership degree. The relative difference of the outbreak size Δ*r* between the standard measure and the Global measure versus the membership degree *om*. The number of overlapping nodes value *on* is equal to 10% of the total number of communities.

**Figure 15:**
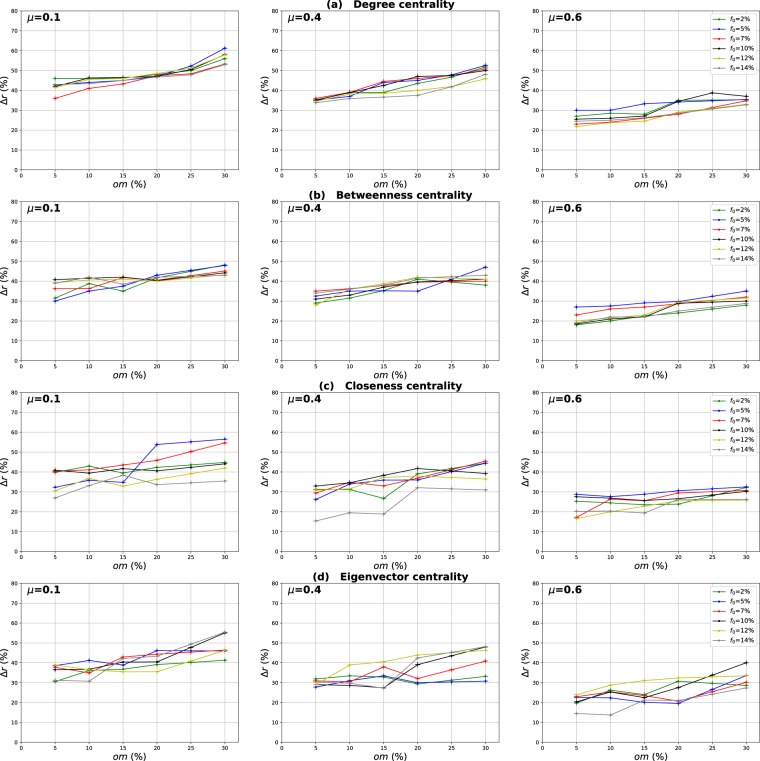
Influence of the community membership degree. The relative difference of the outbreak size Δ*r* between the standard measure and the Modulus measure versus the membership degree *om*. The number of overlapping nodesvalue *on* is equal to 10% of the total number of communities.

Our goal in these experiments is to investigate the impact of the community membership degree of the overlapping nodes on the performance of the Overlapping Modular Centrality. Networks with different community structure strength are used. The proportion of the overlapping nodes is set to 10% of the size of the network. The relative difference of the outbreak size Δ*r* of the local, global and the modulus measures versus the fraction of initial spreaders *f*_0_ is represented in Figs 10–12. It is represented as a function of the community membership degree *om* in Figs 13–15.

We can notice from Figs 10 and 11 that the local and the global measures exhibit the same behavior when the community membership degree varies. We observe in Figs 13 and 14 that the performance of all the centrality measures increases as the membership of the overlapping nodes increases. The performance of the local measure increases by an average value of 10% for Degree centrality in networks with strong and medium community structure, and by 7% in networks with unclear community structure. As reported in Fig. 13, the gain is also around 7% for Betweenness, Closeness and Eigenvector centrality in all the networks with various community structure strength. Figure 14 shows that overall the gain in performance of the global measure is around 5%. Increasing the community membership, the local neighborhood of the overlapping nodes becomes larger. They become more influential in the local network. This is why the local measure performs better if the value of the membership of the overlapping nodes increases. Moreover, the overlapping nodes located in the global network are connected to many foreign communities. So, increasing their community membership, these nodes belong to more communities. Therefore, the epidemic can spread from one community to another through these nodes, affecting a very large number of communities. They can play the role of very influential bridges. This is the reason why the global measure gets more efficient when the value of the membership of the overlapping nodes increases.

Figures 12 and 15 show the results of the same type of experiments for the modulus of the Overlapping Modular Centrality measure. One can see from Fig. 12 that it exhibits the same behavior for the four centrality measures in all the networks under test. Its performance increases while the membership of the overlapping nodes increases as reported in Fig. 15. The gain is around 15% for Degree centrality and 11% for the other tested centrality measures, and this is the case for networks with strong and medium community structure (see the left and the middle panel of Fig. 15). In networks with non-cohesive community structure, the gain is around 10% for all the tested centrality measures (see right panel of Fig. 15). The ranking strategies which are based on a combination of the Overlapping Modular centrality components get also more efficient if we increase the value of the community membership degree of the overlapping nodes.

### Summary

In this section, the Overlapping Modular Centrality extensions of Degree, Betweenness, Closeness and Eigenvector centrality are tested on simulated networks. They are performed at first on networks with different community structure strength having a mixing parameter ranging from 0.1 to 0.6 to test the influence of the community structure on their performance. Results show that for all the centralities, the standard measure performs better than the Global measure while it is less effective than the Local measure in networks with a well-defined community structure. In networks with a medium or weak community structure strength, results show that the Global measure outperforms the standard measure, while the Local measure displays the lower performance. Thus, the performance of the Overlapping Modular centrality components changes with the variation of the proportion of the inter-community links of the network. Additionally, combination-based methods are the most effective measures. Their performance gets even higher if they use properly more information about the community structure. The second investigations aim to show the influence of the proportion of the overlapping nodes on the performance of the Overlapping Modular centrality variations. Results show that by increasing the proportion of the overlapping nodes, the performance of the Overlapping Modular centrality variations increases. Their performance, however, decreases when this proportion becomes very important. In this case, the majority of nodes can belong to several communities which make the network loses its community structure strength. The last part of the experiments aims to show the influence of the community membership degree of the overlapping nodes on the performance of the Overlapping Modular centrality. The experimental results show that whatever the community structure strength of the network, the performance of the extensions of the Overlapping Modular centrality gets higher while increasing the community membership degree of the overlapping nodes.

## Results on Real-World Networks

Results of extensive experiments performed on real-world data originating from various fields (social, collaboration and biological networks) are reported in this section. The aim of this set of experiments is to compare with the results obtained using synthetic networks. Thus, real-world networks with different community structure strength are used. As there is no ground truth data for the networks, the Speaker-Listener Label Propagation Algorithm SLPA is used in order to uncover the overlapping communities. The mixing parameter values estimates are computed according to the uncovered communities. Table 1 reports the estimated values.

**Table 1:** The estimated mixing parameter *μ*, the proportion of the overlapping nodes *on*, and the average number of community memberships per node *m* of the real-world networks.

Network	ego-Facebook	Netscience	ca-GrQc	Princeton	Caltech	Georgetown	Yeast-protein interaction
*μ*	0.075	0.094	0.11	0.25	0.27	0.31	0.47
*on*(%)	8.33	7.84	23.25	7.47	4.55	15.25	33.75
*m*	1.89	2.01	1.65	1.74	1.066	1.41	1.078

These values are computed on the community structure uncovered using the SLPA algorithm.

### Evaluation of the components of the overlapping modular centrality

**Figure 16:**
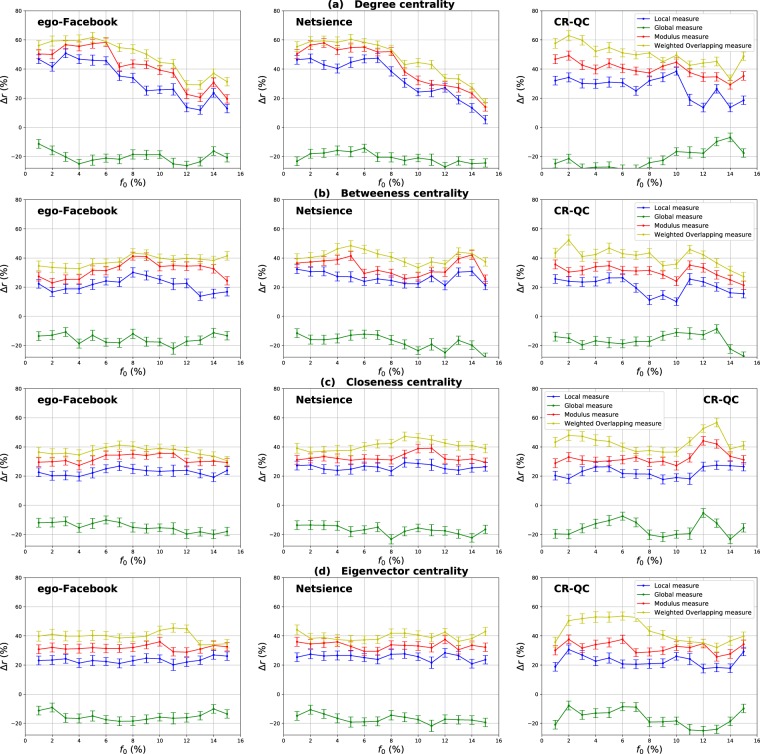
Relative difference of the outbreak size Δ*r* versus the fraction of initial spreaders *f*_0_. Degree (**a**), Betweenness (**b**), Closeness (**c**) and Eigenvector (**d**) centrality measures are compared to their Overlapping Modular extensions using Real-world networks with strong community structure (ego-Facebook, Netscience and ca-GrQc networks). Their mixing coefficient values are equal respectively to 0.02, 0.03 and 0.11.

Results for networks with well-defined community structure (ego-Facebook, Netscience, and ca-GrQc networks) are reported in Fig. 16. The estimated mixing proportion for these networks is equal to 0.075, 0.094 and 0.11 respectively. Figure 16(b) shows that the classical measures outperform always the global component of the Overlapping Modular Centrality measure, while it performs worse than its local component. Let’s consider the Betweenness centrality for example. The gain of its local based-measure over the standard Betweenness has an average value of 25% for the ego-Facebook network, 24% for the Netscience network and 20% for the ca-GrQc network. The global Betweenness is, however, 18%, 17% and 15% less efficient than the standard Betweenness in ego-Facebook, Netscience, and ca-GrQc networks, respectively. In these networks, the communities are sparsely connected with each other. In this case, the chance is high that the epidemic stays confined in the same community and does not propagate to the foreign communities since the contagious area is found in most cases in the core of communities. Therefore, the epidemic spreading may stop even before reaching the nodes located in the community periphery. Thus, there are few chances that the bridges between the communities disseminate the epidemic to other communities. This explains why the local measure always outperforms the global measure. Additionally, one can notice on Fig. 16 that the higher the mixing parameter value the more performing the global component of the Overlapping Modular Centrality measure. This is due to the fact that the number of inter-community links increases while the number of intra-community links decreases.

**Figure 17:**
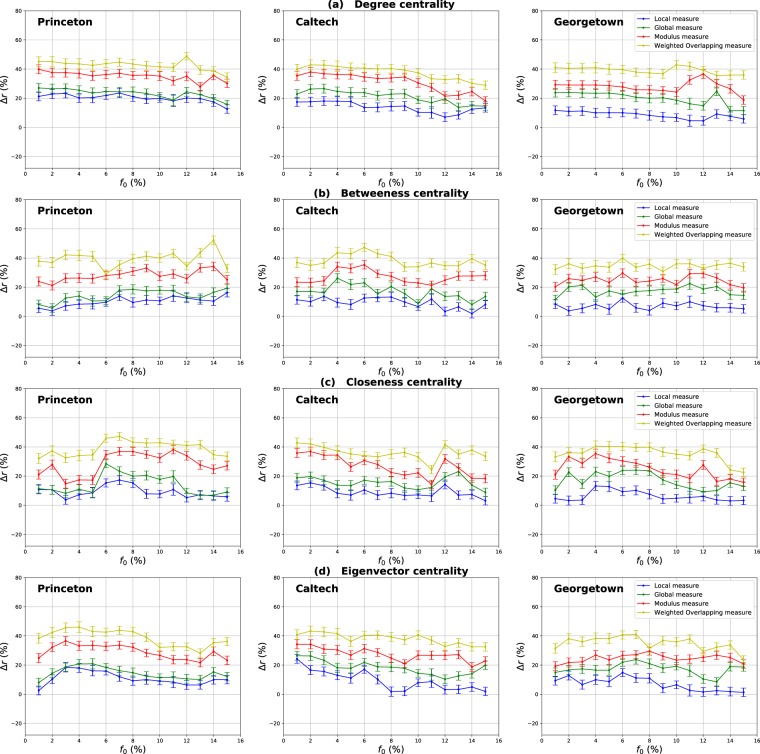
The relative difference of the outbreak size Δ*r* as a function of the fraction of initial spreaders *f*_0_. The Degree (**a**), Betweenness (**b**), Closeness (**c**) and Eigenvector (**d**) centrality measures derived from the Overlapping Modular centrality are compared to the standard counterpart designed for networks with no community structure. Real-world networks with medium community structure (Princeton, Caltech and Georgetown networks) are used. The estimated values of their mixing coefficient is equal respectively to 0.22, 0.26 and 0.3.

Figure 17 shows the same type of results for Princeton, Caltech and Georgetown networks. These networks exhibit a medium community structure strength. Indeed, their estimated mixing parameter values are equal to 0.25, 0.27 and 0.31 respectively. It can be noticed on Fig. 17 that both components of the Overlapping Modular Centrality outperform the traditional measure for all centrality measures under test. Indeed, the local Betweenness performs better than the standard Betweenness with an average gain of 12%, 10% and 9% for Princeton, Caltech and Georgetown networks respectively as it is shown in Fig. 17(b). The global Betweenness, on the other hand, outperforms the traditional Betweenness with an average gain of 14%, 17% and 18% for Princeton, Caltech and Georgetown as it is shown in Fig. 17(b). In these networks, there is a great number of inter-community links connecting nodes from different communities. Thus, these links facilitate the transmission of the epidemic between the communities. In this case, the global influence of the nodes becomes more important than their local influence. Consequently, the global component performs better than the local components for all the centrality measures. One can see also on Fig. 17 that the performance of the global component is close to the performance of the local component in the Princeton network. It increases in Caltech and Georgetown networks that have higher mixing parameter values. Thus, the global influence of nodes grows with the number of inter-community links.

**Figure 18:**
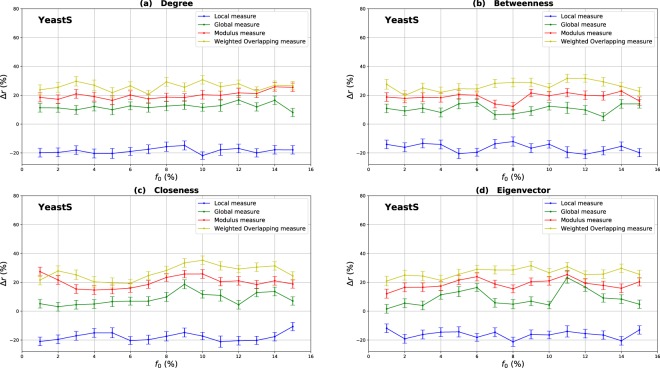
The relative difference of the outbreak size Δ*r* as a function of the fraction of initial spreaders *f*_0_. The Degree (**a**), Betweenness (**b**), Closeness (**c**) and Eigenvector (**d**) centrality measures derived from the Overlapping Modular centrality are compared to the standard counterpart designed for networks with no community structure. A real-world network (YeastS) with weak community structure is used. The estimated value of its mixing coefficient is equal to 0.47.

Experimental results with the Yeast-protein interaction network characterized by a loose community structure are reported in Fig. 18. One can see that the relative difference of the outbreak size Δ*r* is always positive for the global measure, while it is always negative for the local measure. Thus, the standard measure outperforms the local measure, and it is less performing than the global measure for all the centrality measures. The gain of the global Betweenness measure over the standard Betweenness is around 16%. The local Betweenness measure, on the other hand, is 17% less efficient than the standard Betweenness as reported in Fig. 18(b). In this network, there is a large amount of inter-community links, so nodes have a big global influence. This is the reason why the global measure always outperforms the local measure. Moreover, the gain of the global measure gets smaller in this network. This is due to the weak community structure strength of this network, which leads to a decrease in the effectiveness of the modular-based measures.

### Evaluation of the ranking methods based on a combination of the components of the overlapping modular centrality

Experimental results of the evaluation of the ranking measures based on combining the Overlapping Modular Centrality components are reported in Figs 16–18. They are quite similar to the ones observed with synthetic networks for all the centrality measures under test. Indeed, both combination strategies always outperform the traditional measure and each component of the Overlapping Modular Centrality in any case. The modulus of the Overlapping Modular Betweenness centrality performs better than the standard measure with an average gain that ranges between 39% and 47% in networks with well-defined community structure (ego-Facebook, Netscience and cr-GrQc), between 30% and 35% in networks with community structure of medium strength (Princeton, Caltech and Georgetown) and around 24% in the Yeast-protein interaction network that has a weak community structure. The weighted Overlapping Modular measure of the Betweenness centrality is overall the most efficient.

As expected, using a simple combination strategy allows more effective results. In addition, using relevant information about the communities allows obtaining even better results, as it is in the case of the Weighted Overlapping Modular measure. Indeed, this measure uses more information about the community structure such as the proportion of inter-community links of each module in the network. It incorporates also the size of communities as well as the number of foreign communities attached to each node. That explains the effectiveness of this strategy compared to the simple modular Combination. Furthermore, we can also notice that the best results are obtained for networks with a well-defined community structure (ego-Facebook, Netscience and ca-GrQc). The performance of the combination strategies decreases with the community structure strength as observed in the case of Yeast-protein interaction network.

### Comparison with the alternative community-based methods

**Figure 19:**
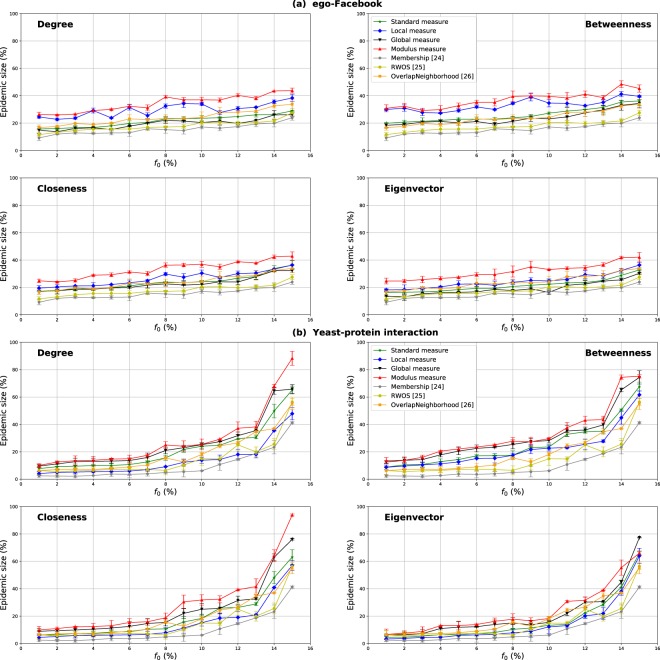
The epidemic size as a function of the fraction of initial spreaders *f*_0_. The standard and the modular variations of Degree, Betweenness, Closeness and Eigenvector centrality measures as well as some alternative measures (Membership, RWOS and Overlap Neighborhood) are tested on two real-world networks of different community structure strength.

The epidemic size as a function of the proportion of the initial spreaders for the Overlapping Modular Centrality (modulus, local component, global component) and their various classical counterparts (Degree, Closeness, Betweenness, Eigenvector) is illustrated in Fig. 19. The performance of the alternative methods (i.e., Membership, Random-Walk Overlap Selection *RWOS* and OverlapNeighborhood) is also represented in this figure. The results for the ego-Facebook network are reported in Fig. 19(a), while they are reported in Fig. 19(b) for the Yeast-protein interaction network. We choose these two networks because ego-Facebook has a well-defined community structure while Yeast-protein interaction has a loose community structure. The aim of this section is to compare the performance of all the variations of the Overlapping Modular Centrality with the alternative methods designed for modular networks (refer to the Materials and Methods section).

Results show that the epidemic size increases with the proportion of the initial spreaders. This is true for all the tested measures and for the two networks. However, this evolution is moderate in ego-Facebook network while it is faster in Yeast-protein interaction network. In the first network, the communities are well-separated, the epidemics cannot then propagate very easily between its modules. In both networks, the OverlapNeighborhood performs, in most cases, as well as the standard Degree centrality. It even surpasses, in some cases, the performance of standard Betweenness centrality. Also, it outperforms the standard Closeness and Eigenvector centralities and the other alternative methods (Membership and *RWOS*). The OverlapNeighborhood selects randomly the neighbors of the overlapping nodes. Indeed, overlapping nodes are more likely to be connected to hubs in each community to which they belong. This is the reason why it performs as well as the standard Degree centrality. Moreover, for both networks, one can see in Fig. 19 that the curves of *RWOS* and the Membership are always located at the bottom of all the figures. They perform poorly. These two measures do not use as much information about the network topology as compared to the other measures. That is why they exhibit lower performance.

Additionally, in ego-Facebook network, the local component of the Overlapping Modular Centrality outperforms the alternative measures. The global component is, however, more efficient than the alternative methods in Yeast-protein interaction network. The former network has a well-defined community structure. Nodes with high intra-community connections are more susceptible to infect many nodes of the network. That’s what makes the Local measure very efficient in ego-Facebook since it targets nodes of different nature (overlapping and non-overlapping) with high local influence. The second network, on the other hand, has a loose community structure. Nodes with high inter-community links are more likely to have the highest influence on this type of networks. This is the reason why the performance of the global measure outperforms alternative methods. Furthermore, the modulus is the most influential measure in both networks and for all the centrality measure under test. This combination-based method considers the local and global influence of both non-overlapping and overlapping nodes. Therefore, combination methods can highlight the key nodes in the diffusion process.

### Influence of the community detection algorithms

#### Comparison with alternative algorithms

**Figure 20:**
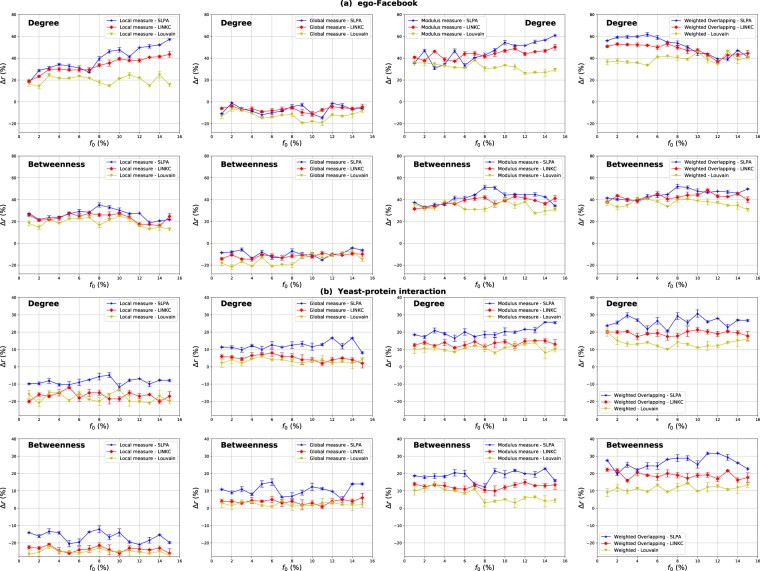
The relative difference of the outbreak size Δ*r* as a function of the fraction of initial spreaders *f*_0_. The Degree, Betweenness centrality measures derived from the Overlapping Modular centrality are compared to their standard counterparts. The measures are performed on ego-Facebook network in (**a**) and Yeast- protein interaction network in (**b**) for the SLPA, LINKC and Louvain algorithms.

We report in this section a set of experiments on ego-Facebook and Yeast-protein interaction networks using Link Communities (LINKC)^33^ and Louvain^34,35^ algorithms. The first algorithm is used to test the robustness of the results if a different overlapping community detection method is chosen. The second algorithm is used to compare the Overlapping Modular centrality with the Modular centrality since Louvain algorithm is designed for non-overlapping communities. Figure 20 represents the relative difference of the outbreak size between the Overlapping Modular centrality extensions (Degree and Betweenness centrality) and their classical counterparts. These measures are computed on two real-world networks: the ego-Facebook network characterized by a well-defined community structure, and the Yeast-protein interaction network that has a loose community structure. The estimated values of the mixing parameter and the proportion of the overlapping nodes are reported in Table 2.

**Table 2:** The estimated mixing parameter *μ*, the proportion of the overlapping nodes *on*, and the average number of the community membership *m* in ego-Facebook and Yeast-protein interaction networks.

Network	Metric	Detection algorithm		
		SLPA	LINKC	Louvain
ego-Facebook	*μ*	0.075	0.096	0.03
	*on*(%)	8.33	7.09	0
	*m*	1.89	1.37	0
Yeast-protein interaction	*μ*	0.47	0.51	0.49
	*on*(%)	33.75	6.03	0
	*m*	1.078	1.64	0

It can be inferred from Fig. 20, that for both algorithms (LINKC and Louvain), the local component, global component and the modulus of the Overlapping Modular Centrality exhibit overall the same behavior than with the SLPA algorithm. In networks with well-defined community structure (e.g., ego-Facebook network), the local component performs always better than the classical one with an average gain of 33% and 20% for Degree centrality, while the gain is around 23% and 19% for Betweenness centrality when employing LINKC and Louvain algorithms respectively. The average gain is around 37% and 26% in the case of the SLPA algorithm for both centrality measures. The standard measure, on the other hand, outperforms the global component for all the proportions of the initial seeds. Additionally, the overall gain of the modulus of the Overlapping Modular Centrality is around 44% and 32% for Degree centrality, while it is around 38% and 34% for Betweenness centrality when using LINKC and Louvain algorithms respectively. The average gain, however, is around 46% and 42% for both centrality measures when using SLPA. Thus, if we compare the different overlapping community detection methods, it appears that the Overlapping Modular centrality extension based on the SLPA algorithm is slightly more effective. Indeed, for both algorithms, the same mixing parameter values and the proportion of overlapping nodes are quite similar. Therefore, in networks with a well-defined community structure, they uncover quite the same community structure. This is why the performance of the Overlapping Modular centrality displays roughly the same behavior. However, the variations of the Overlapping Modular Centrality exhibit the lowest performance when Louvain algorithm is used. This shows the importance of the overlapping nodes in the diffusion process.

In networks with a loose community structure (e.g., Yeast-protein interaction network), the standard measure is always outperforming the local component of the Overlapping Modular Centrality. The global component, however, performs better than the Standard centrality with an average gain of 5% and 3% for Degree centrality, while the gain is around 4% and 2% for Betweenness centrality when employing LINKC and Louvain algorithms respectively. The average gain is around 12% and 10% in the case of the SLPA algorithm for both centrality measures. The overall gain of the Modulus measure is around 14% and 10% for Degree centrality, while it is around 13% and 7% for Betweenness centrality when using LINKC and Louvain algorithms respectively. In addition, the gain is around 20% and 19% for both centrality measures respectively when the SLPA algorithm is used. In this network, the performance of the Overlapping Modular Centrality components for the SLPA algorithm is higher than the ones obtained when the LINKC algorithm is employed. Indeed, the SLPA algorithm has a relatively smaller mixing parameter and a higher proportion of the overlapping nodes. As expected, the performance of the variations of the Overlapping Modular Centrality increases as the proportion of the overlapping nodes increases. These results are online with the ones based on synthetic networks experiments. Thus, it suggests that SLPA is more accurate than LINKC algorithm. The lowest performance, however, is obtained using the Louvain algorithm even-though it has a smaller mixing parameter as compared to LINKC algorithm. This algorithm does not consider the overlapping nodes. Thus, we can conclude that taking into account the influence of the overlapping nodes enhances the performance of the Modular centrality components.

Moreover, the right panel of Fig. 20 reports the performance of the Weighted Overlapping and the Weighted non-overlapping measures for Degree and Betweenness centralities. The parameters of the Weighted Overlapping measures are computed using either SLPA or LINKC and are respectively called Weighted Overlapping SLPA and Weighted Overlapping LINKC. The parameters of the Weighted non-overlapping measure are computed using the Louvain algorithm. In ego-Facebook network, the Weighted Overlapping SLPA outperforms the Weighted non-overlapping measure with an average gain of 10% and 8% respectively for Degree and Betweenness centrality measures. The Weighted Overlapping LINKC performs also better than the Weighted non-overlapping measure with an average gain of 7% and 6% for Degree and Betweenness centrality measures. In the Yeast-protein interaction network, the Weighted Overlapping SLPA performs 13% and 14% higher than the Weighted non-overlapping measure for Degree and Betweenness centralities respectively. In addition, the Weighted Overlapping LINKC outperforms the Weighted non-overlapping measure with an average gain of 7% for both Degree and Betweenness centralities. As expected, in both networks, the Weighted Overlapping measures are more effective than the Weighted non-overlapping measure. Furthermore, the Weighted Overlapping SLPA has the best performance. Therefore, taking into account the overlaps among communities is substantial in order to highlight the influential spreaders in modular networks.

Overall, the results of this set of experiments show that the performance of the Overlapping Modular Centrality is influenced by the variations of the uncovered community structure. Whatever the community structure strength, the SLPA and LINKC detection algorithms present most of the time a better performance as compared to Louvain algorithm. Therefore, the proposed centrality exhibits a higher performance when an overlapping community detection is used. That shows the major role that plays the overlapping nodes in terms of disseminating epidemics between the communities.

#### Comparison with ground truth data

We also perform a set of experiments to compare the performance of the Overlapping Modular Centrality measures obtained on a network where the set of communities is known with the case where the communities are discovered through a community detection algorithm. Two synthetic networks with known community structure are used: A LFR network with a well-defined community structure (small mixing parameter value µ = 0.1), and one with a loose community structure (large mixing parameter value µ = 0.6). The SLPA algorithm is used to uncover the community structure of these networks due to its effectiveness as compared to the LINKC algorithm. The mixing parameter, the proportion of the overlapping nodes, the number of communities are computed once the community structure is revealed using the SLPA algorithm. The estimated values are listed in Table 3. Furthermore this table contains the Normalized Mutual Information (NMI) shared by the two community structure (ground truth, uncovered by SLPA). Note that, the more similar the structures the higher the NMI value.

**Table 3:** *μ* is the estimated mixing parameter. *on* is the proportion of the overlapping nodes.

Network	Metric	Ground truth	SLPA
LFR (*μ* = 0.1)	*μ*	0.1	0.141
	*on*(%)	10	12
	*N*_*c*_	110	130
	*NMI*	0.601	
LFR ($\mu$ = 0.6)	*μ*	0.6	0.546
	*on*(%)	10	6.2
	*N*_*c*_	74	197
	*NMI*	0.178	

*N*_*c*_ the number of communities membership. *NMI* is the Normalized Mutual Information shared by the two community structure (ground truth, uncovered by SLPA).

**Figure 21:**
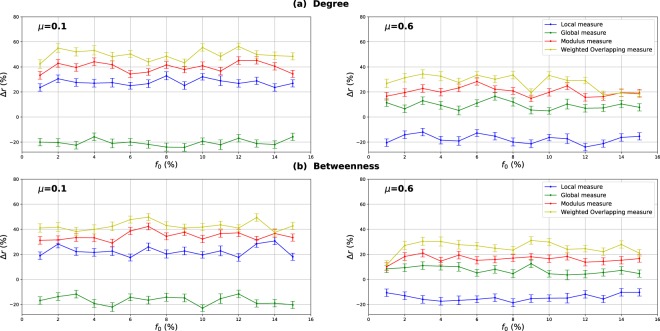
The relative difference of the outbreak size Δ*r* as a function of the fraction of initial spreaders *f*_0_. The Degree (**a**), Betweenness (**b**) centrality measures derived from the Overlapping Modular centrality are compared to their standard counterpart. Two synthetic networks (LFR) with respectively well-defined ($\mu =0.1$) and loose community structure ($\mu =0.6$) are used. The measures are performed using the SLPA detection algorithm.

Results for the network with well-defined community structure (µ = 0.1) show that the performance of the Overlapping Modular centrality variations exhibits the same behavior for both SLPA and ground truth data. The standard measure outperforms always the Global measure while it performs less than the Local measure. In addition, combination-based measures are the most effective measures. It is also noticed from Fig. 21(a) that the performance of all the measures is lower with an average value ranging from 2% to 3% for SLPA algorithm. Indeed, the estimated values of the proportion of the overlapping nodes, the number of the partitions and the mixing parameter are relatively close to the values generated by the LFR algorithm. Furthermore, the NMI parameter has a high value. Thus, the SLPA algorithm can roughly detect the same set of communities than the ones defined by the LFR algorithm. This is why the performance is quite similar.

The results of the LFR network with a loose community structure (µ = 0.6) are illustrated in Fig. 21(b). In this figure, the performance of the standard measure is always lower than the Global measure and higher than the Local measure, while the combination-based methods show the best performance for SLPA as in the case where the community structure of the LFR network is known. However, the Overlapping Modular Centrality variations have a lower performance with an average value of 7% for SLPA algorithm. The estimated parameters for the SLPA algorithm are quite different than the ones generated by the LFR algorithm. Moreover, the NMI value is very small. Therefore, the communities revealed by the SLPA are quite different than the set of communities generated by the LFR algorithm. This is why the performance of the extensions of the Overlapping Modular centrality is lower when the SLPA algorithm is used. This experiment shows that when the communities are discovered through a community detection algorithm, the performance of the Overlapping Modular centrality variations may vary as compared to the case when the set of communities are known. The highest variations are noticed in the case of networks with non-cohesive community structure. Indeed, the weaker the community structure the more difficult it is to uncover it.

### Summary

In this paper, we performed a series of experiments on real-world networks of various nature covering all the range of community structure strength. The SLPA algorithm is used to uncover the overlapping communities of the network. Results show that the performance of the Overlapping Modular centrality versions of the four tested centralities exhibits the same behavior as for synthetic networks. Indeed, the Local component shows its best performance in networks with well-defined community structure, while the Global component shows its best performance in networks with medium and weak community structure strength. Moreover, combination-based measures outperform all the other measures in all the different types of networks. These results corroborate the conclusions we made with the synthetic networks. The second part of the experiments aims to compare the Overlapping Modular centrality together with their classical counterparts with some alternative measures (Membership, RWOS and OverlapNeighborhood). Experimental results show the OverlapNeighborhood performs, in most cases, as well as the standard Degree centrality and surpasses in some cases the standard Betweenness centrality. The other alternative measures (RWOS and the Membership) display a lower performance. This is due to their use of limited information about the network topology as compared to the other measures. Another set of experiments is performed to show the influence of the community detection algorithm choice on the performance of the Overlapping Modular centrality. We use the LINKC algorithm to test the robustness of the results if a different overlapping community detection algorithm is chosen. We used also the Louvain algorithm which is designed to uncover non-overlapping communities in order to compare the Overlapping Modular centrality with the Modular centrality. In networks with well-defined community structure, results show that the performance of the Overlapping Modular centrality does not vary significantly when the LINKC algorithm is used. This is due to the fact that both algorithms reveal about the same set of communities. However, the performance of the Overlapping Modular centrality variations decreases for LINKC algorithm in networks with loose community structure. In this case, the community detection algorithms uncover different sets of communities. Furthermore, whatever the community structure strength, the Overlapping Modular centrality (using SLPA or LINKC) outperforms the Modular centrality (using Louvain). Therefore, the overlapping nodes play a major role in terms of disseminating epidemics between the communities.

## Conclusion

A new framework allowing to extend classical centrality measures to overlapping modular networks is introduced. Our approach is inspired by the idea that one needs to consider two types of influences for a node in a modular network: a local influence on the nodes belonging to their communities exerted through the intra-community links, and a global influence on the nodes of the remaining communities that goes through the inter-community links. Therefore, in overlapping modular networks, centrality measures need to be represented by a two-dimensional vector that we call the “Overlapping Modular centrality”. One component measures the local influence of the node, while the other measures its global influence. A series of experiments have been performed in order to test the effectiveness of the Overlapping Modular centrality extensions as compared to their counterpart defined for networks with no community structure. Considering the most influential centrality measures (Degree, Betweenness, Closeness, Eigenvector), the local and global components have been evaluated separately. Additionally, a straightforward combination of both components (modulus of the two-dimensional vector) has been tested. Another combination based method (Weighted Overlapping Modular centrality) that uses additional knowledge about the community structure topology has been also evaluated. Experiments have been conducted on synthetic and real-world networks using the SIR epidemic model. Results show that the spreaders identified by the proposed approach are more influential than those targeted by the centrality measures designed for non-modular networks. Furthermore, investigations conducted on synthetic networks show that the performance of the Overlapping Modular Centrality increases with the proportion of the overlapping nodes and their community membership degree. Comparisons with alternative centrality measures designed for modular networks are in favor of the proposed framework. Additionally, experiments performed on real-world networks with various community detection algorithms show that the Overlapping Modular Centrality is very robust to community structure variations, and it demonstrates the importance of the overlapping nodes in the epidemic spreading process.

## Materials and Methods

### SIR simulations

The performance of the centrality measures is evaluated based on the SIR epidemiological model^36,37^. Each node can be in one of the three states: Susceptible (S), Infected (I) and Recovered (R). Starting with all the nodes in the susceptible state, a given proportion *f*_0_ of the top-ranked nodes according to the centrality measure under test are infected. At each iteration, the infected nodes can transmit the infection to their susceptible neighbors with the infection rate *λ*. Infected nodes can recover with a recovery rate *γ* and they cannot be infected again. The process stops when there are no more infected nodes. The total number of recovered nodes is recorded to measure the effectiveness of the immunization strategy. The higher this value, the more efficient the method.

The value of the transmission rate *λ* is chosen to be greater than the network epidemic threshold *λ*_*th*_ in order to better characterize the spreading capability, it is defined as^38^:

2 $${\lambda }_{th}=\frac{\langle k\rangle }{\langle {k}^{2}\rangle -\langle k\rangle }$$

where $\langle k\rangle$ and $\langle {k}^{2}\rangle$ are respectively the first and second moment of the degree distribution. The epidemic threshold values *λ*_*th*_ of the networks used in this paper are reported in Table 5. The same transmission rate value ($\lambda =0.1$) is used in all the experiments. It is higher than the epidemic threshold *λ*_*th*_ values of all the data collection used in this work. The value of the recovery rate *γ* is set to 0.1. This small value is chosen in order to give more chances to infected nodes to infect their neighbors (with the probability *λ*) before turning to the recovered status.

### Generation of synthetic networks

The LFR (Lancichinetti, Fortunato and Radicchi) algorithm^30^ is used to produce synthetic networks. It guarantees networks with realistic features^39^. It allows also to control various parameters, such as the mixing parameter *μ*. This parameter controls the strength of the community structure. It is defined as the ratio of the number of external neighbors of a node to the total degree of the node. As this value becomes smaller, the community structure becomes stronger (a small proportion of the inter-community links), making community identification an easy task. The LFR algorithm allows to control the number of overlapping nodes *on* and their membership number *om* (the number of communities to which they belong). Previous studies showed that the typical value of the degree distribution exponent usually ranges from 2 to 3 in real-world networks. We choose some consensual values for the parameters that are reported in Table 4.

**Table 4:** LFR network parameters.

Number of nodes	4000
Average degree	10
Maximum degree	20
Exponent for the degree distribution	2.7
Exponent for the community size distribution	2.5
Mixing parameter	0.1, 0.4, 0.6
Proportion of overlapping nodes	[5, 50]%
Membership degree	[5, 30]%

### Empirical networks

Some topological properties such as transitivity and degree correlation cannot be controlled in networks generated by the LFR algorithm. They may differ considerably from those observed in real networks^39,40^. Consequently, one must perform experiments with real-world networks in order to validate the synthetic network’s results. We choose to use networks originating from various fields such as social networks, collaboration networks, and biological networks in order to have exhaustive results. Experiments are performed on undirected and unweighted networks and only on their largest connected component. In order to uncover the overlapping community structure of these networks, the Speaker-Listener Label Propagation Algorithm SLPA^41^ is used. We choose this algorithm because it is one of the highly ranked algorithms according to the Normalized Mutual Information (NMI) measure^42^. Furthermore, previous studies show that it is a good compromise when used on networks of various origin^31^. For more information about the networks report to^12,43–46^.

- **Social networks:** We use four Facebook networks: the Facebook friendship network at 3 US universities (Caltech, Princeton and Georgetown) collected by Traud *et al*.^44^ and the ego-Facebook network based on a survey of users of the Facebook app.^43^. Nodes represent individuals and there is a link between two nodes when two individuals are friends online.
- **Collaboration network:** Two collaboration networks are used. General Relativity and Quantum Cosmology collaboration network GR-QC^46^ covers scientific collaborations between authors of papers submitted to the General Relativity and Quantum Cosmology category. There is an edge between two authors if they co-authored a paper.
  Netscience^12^ is a co-authorship network of scientists whose research centers on network theory and experiment. Nodes represent authors and edges join individuals that co-authored a paper.
- **Biological network:** The Yeast-protein interaction^45^ is a network formed of protein interactions contained in yeast. A node represents a protein and an edge represents a metabolic interaction between two proteins.

Table 5 reports the basic topological properties of real-world networks.

**Table 5:** Basic topological properties of the real-world networks.

Network	*N*	*E*	〈*k*〉	*k*_*max*_	*C*	*λ*_*th*_
ego-Facebook	4039	88234	43.69	1045	0.605	0.009
Netscience	1589	2742	3.45	34	0.74	0.052
ca-GrQc	4158	13428	5.53	81	0.529	0.059
Princeton	5112	28684	88.93	628	0.298	0.006
Caltech	620	7255	43.31	248	0.443	0.012
Georgetown	7651	163225	90.42	1235	0.268	0.006
Yeast-protein interaction	1870	2277	2.43	56	0.051	0.095

*N* is the number of nodes, *E* is the number of edges. 〈*k*〉 is the average degree, *k*_*max*_ is the max degree. *C* is the average clustering coefficient. *λ*_*th*_ is the epidemic threshold.

### Alternative centrality measures designed for modular networks

The centrality measures presented below are the most efficient for ranking nodes according to various community structure properties. Note that purely random strategies are not considered because their performance is generally well-below the deterministic strategies based on node ranking.

#### Membership centrality

This measure^24^ counts the number of communities of a node. If the membership of a node *v*_*i*_ is greater than 1 it belongs to an overlapping region. Experimental results using the SIR model have shown that this centrality measure outperforms degree, coreness and betweenness centrality measures in networks with dense communities under high infection rates.

#### Random-walk overlap selection (RWOS)

This method proposed by F. Taghavian *et al*.^25^ selects the overlapping nodes according to a random walk. It starts by defining the list of the overlapping nodes extracted from the communities. Then, a random-walk is run from a node selected at random. If a visited node is an overlapping node, it is then considered as an influential spreader, otherwise, the random-walk proceeds. The aim of this method is to target the high degree overlapping nodes. SIR simulations have shown that RWOS performs better than the most efficient local methods using a limited amount of information such as CBF^14^ and BHD^15^. In addition, it performs nearly as good as Degree and Betweenness centrality especially in networks with well-defined community structure and with high overlap membership values.

#### OverlapNeighborhood

This method^26^ targets the immediate neighbors of overlapping nodes as the top influential spreaders. Its main objective is to select the most highly connected nodes using a limited amount of information at the community level. Indeed, there is a high probability that nodes with very high connections are neighbors to overlapping nodes since they are part of more than one community. Experiments revealed that it outperforms CBF, BHD and RWOS methods. Furthermore, it performs better or as well as Degree and Betweenness centrality measures without the need to know the overall network structure.

#### Modular centrality

In a previous work^29^, we introduced a framework to extend centrality measures designed for non-modular networks to networks with non-overlapping communities. In this case, centrality is represented by a two-dimensional vector called the Modular centrality. One dimension measures the local influence of a node while the other measures its global influence. Results have shown that derived ranking measures based on combining the two components of the Modular centrality outperform the traditional centrality measures. Better results have been obtained by using more information about the topological properties of the communities. However, this measure is not appropriate for modular networks containing nodes that can belong to multiple communities.

### Combining the components of the overlapping modular centrality

In order to obtain a ranking list based on the Overlapping Modular Centrality two-dimensional vector, a ranking measure need to be derived. We consider a straightforward combination and a more sophisticated one that integrates additional topological properties of the community structure. At first we propose to rank the nodes according to the modulus of the Overlapping Modular Centrality components. The modulus *r* of the Overlapping Modular centrality vector *B*_*OM*_ of a node *v*_*i*_ is defined as follows:

3 $$r({v}_{i})=\parallel {B}_{OM}({v}_{i})\parallel =\sqrt{{({\beta }_{L}({v}_{i}^{y}))}^{2}+{({\beta }_{G}({v}_{i}^{q}))}^{2}}\,y\in \{1,\ldots ,z\}\,{\rm{and}}\,q\in \{1,\ldots ,p\}$$

where *β*_*L*_ is the Local measure and *β*_*G*_ is the Global measure.

Note that no knowledge is used about the communities topological properties. We consider the so-called “Weighted Overlapping Modular Centrality”. This second measure is a weighted linear combination of the local and the global components of the Overlapping Modular centrality that incorporates more knowledge about the communities.

Let’s consider an epidemic starting at the core of a community. It can propagate easily to the network if the community is tightly connected to the others, while it may stay in the community if this one is well isolated. Therefore, the diffusion capacity of a node is dependent on its position in its community and also to the interactions that its community has with the rest of the network. Based on this idea, we propose a ranking measure that can adapt to the node environment. Its local component is weighted by the community size in order to give more weight to the hubs located in the biggest communities. The global component is weighted by the number of neighboring communities. The goal is to target the nodes allowing to reach a great number of communities through numerous connections. The Weighted Overlapping Modular centrality measure for a given node *v*_*i*_ is then given by:

4 $${\beta }_{w}({v}_{i})=h({v}_{i}^{y})+b({v}_{i}^{q})\,y\in \{1,\ldots ,z\}\,{\rm{and}}\,q\in \{1,\ldots ,p\}$$

where

5 $$h({v}_{i}^{y})=(1-\rho )\,\ast \,\frac{{S}_{{M}_{y}}}{N}\,\ast \,{\beta }_{L}({v}_{i}^{y})\,y\in \{1,\ldots ,z\}$$

and

6 $$b({v}_{i}^{q})=\rho \,\ast \,\frac{\alpha ({v}_{i}^{q})}{{n}_{c}}\,\ast \,{\beta }_{G}({v}_{i}^{q})\,q\in \{1,\ldots ,p\}$$

where ${S}_{{M}_{y}}$ is the size of the local neighborhood *M*_*y*_ (${S}_{{M}_{y}}=card({M}_{y})$). *N* is the size of the network. $\alpha ({v}_{i}^{q})$ is the number of the neighboring communities connected to the node ${v}_{i}^{q}$ while *n*_*c*_ is the total number of communities in the network. $\rho$ is the fraction of the inter-community links of the local neighborhood *M*_*y*_. It is defined as follows:

7 $$\rho =\frac{{\sum }_{{v}_{i}^{y}\in {M}_{y}}{k}^{{inter}}({v}_{i}^{y})}{{\sum }_{{v}_{i}^{y}\in {M}_{y}}k({v}_{i}^{y})}\,y\in \{1,\ldots ,z\}$$

where $k({v}_{i})$ is the degree of node *v*_*i*_ and ${k}^{inter}({v}_{i})$ represents the number of inter-community links of node *v*_*i*_.

This measure performs as follows:

- If the node *v*_*i*_ belongs to a local neighborhood (or a community in the case of a non-overlapping node) in a network with a well-defined community structure, then the value of $\rho$ is small. In this case, the local measure gets more weight, and the Weighted Overlapping Modular measure favors the community hubs.
- If the node *v*_*i*_ belongs to a local neighborhood (or a community in the case of a non-overlapping node) in a network with a loose community structure, then $\rho$ has a high value. More importance is given to the global measure and the Weighted Overlapping Modular measure favors the nodes with a high amount of inter-community links that can diffuse the epidemic all over the network.

## Data Availability

The datasets used in this article are all publicly available and cited in the references.
